# Brain functional imaging contributions in osteoarthritis-related pain: A viewpoint

**DOI:** 10.1016/j.ocarto.2024.100554

**Published:** 2024-11-28

**Authors:** Camille Fauchon, Marie Binvignat, Francis Berenbaum, Philip G. Conaghan, Roland Peyron, Jérémie Sellam, the Going Inside Osteoarthritis-Related Pain Phenotyping (GO-PAIN) network members, Françoise Alliot-Launois, Nadine Attal, Francis Berenbaum, Marie Binvignat, Philip Conaghan, Alice Courties, Niels Eijkelkamp, Camille Fauchon, Rinie Geenen, Ida K. Haugen, Yves Henrotin, Kalle Kisand, Margreet Kloppenburg, Eva Kosek, Liisa Kuhi, Sylvain Mathieu, Céline Mathy, Ali Mobasheri, Stanislas Moumbe Talla, Patrick Omoumi, Serge Perrot, Roland Peyron, Simo Saarakkala, Alain Saraux, Hans-Georg Schaible, Jérémie Sellam

**Affiliations:** a University of Clermont Auvergne, CHU Clermont-Ferrand, Inserm, Neuro-Dol, Clermont-Ferrand, France; b Department of Rheumatology, Saint-Antoine Hospital, Assistance Publique–Hôpitaux de Paris (AP-HP), Paris, France; c Centre de Recherche Saint-Antoine (CRSA) Inserm UMRS-938, Sorbonne Université, Paris, France; d Leeds Institute of Rheumatic and Musculoskeletal Medicine, University of Leeds, Leeds, UK; e Université Jean Monnet, CHU Saint-Etienne, Inserm UMR-1028, CRNL, NeuroPain, Saint-Etienne, France; f NIHR Leeds Biomedical Research Centre, Leeds Teaching Hospitals NHS Trust, Leeds, UK

**Keywords:** Osteoarthritis, Brain imaging, Biomarkers, Chronic pain

## Abstract

**Objective:**

Neuroimaging investigations are critical to provide a more direct assessment of brain disturbances associated with osteoarthritis (OA)-related pain, and to better understand its pathophysiology to develop new treatment strategies. This viewpoint aims to summarize the importance of the brain in OA pain.

**Method:**

A European working group on pain in osteoarthritis GO-PAIN (Going Inside Osteoarthritis-related Pain Phenotyping) has been created to work on a global assessment of the OA-related pain. Relevant scientific literature was evaluated, summarized and discussed to expose advances in functional brain alterations related-to OA pain.

**Results:**

Findings of neuroimaging studies are highly heterogenous and based on small sample size, but some key brain alterations associated with OA pain can be identified across experiments. A systematic literature review conducted by Hall and colleagues (2023) found lower activity, connectivity, and grey matter volume in the right anterior insula in patients with OA than in healthy controls. Other works also pointed out that activity of specific brain regions could serve as a potential surrogate biomarker, but several limitations and confounding factors needs to be addressed.

**Conclusions:**

Brain functional imaging provides opportunities to accurately address an OA-related pain endophenotype. To encompass limitations and fill the gaps from the previous studies, we propose a blueprint for the next 5 years and stimulate ideas for others working in the field.

## 1 Osteoarthritis-related pain: from the joint to the brain

Pain is the main symptom reported by people with osteoarthritis (OA), driving poor quality of life and providing the main driver to seek care. Since treatment of OA is currently symptom-focused, OA-related pain orchestrates therapeutic decision-making. Despite this, the mechanisms underlying OA pain remain poorly understood.

Conventionally, pain in humans is assessed using visual analogue scales (VAS) or numeric rating scales, or combined algo-functional patient-reported outcomes (PROs). Such tools are easy to use but are not able to discriminate the complexity encompassed by this symptom. Indeed, OA-related pain combines several mechanisms that include pain sensitization, nociceptive, inflammatory and neuropathic pain and may be influenced by psychosocial comorbidities and socioeconomic circumstances [1,2], but also immune alterations, hormonal changes, sleep impairments, and (epi)genetic features. Nociceptive pain is classically due to non-neural joint tissue damage, and joint imaging studies have emphasized the prominent role of innervated tissues such as synovium and subchondral bone [1].

Inflammation is a key feature of OA-related pain (see for review [3]). Experimental data have shown that subchondral bone, cartilage and synovium can be the source of inflammatory mediators implicated in the initiation and perpetuation of the OA process and illustrating the “whole-joint disease” concept (see Ref. [4] for details). Beside inflammatory pain, several works have also pointed out that neuropathic pain may also occur at least in a subset of the OA population and its precise mechanism is not clear [5]. In contrast to inflammatory or nociceptive pain, which is caused by actual tissue damage or potentially tissue-damaging stimuli, neuropathic pain is therefore produced by damage to the peripheral or the central somatosensory nervous system. Some people with knee OA use descriptors such as burning, tingling, numbness, and pins and needles to characterize their knee symptoms. Such descriptors suggest that neuropathic pain may contribute to the OA pain experience, although specific nerve lesions in the somatosensory system are not always clearly identified leading to the use of the term “neuropathic-like pain”. Both a lesion or disease confirmed with a diagnostic test like electroneuromyography or MRI, and sensory signs related to the area that this lesioned structure innervates are necessary to confirm definite neuropathic pain.

**Fig. 1:**
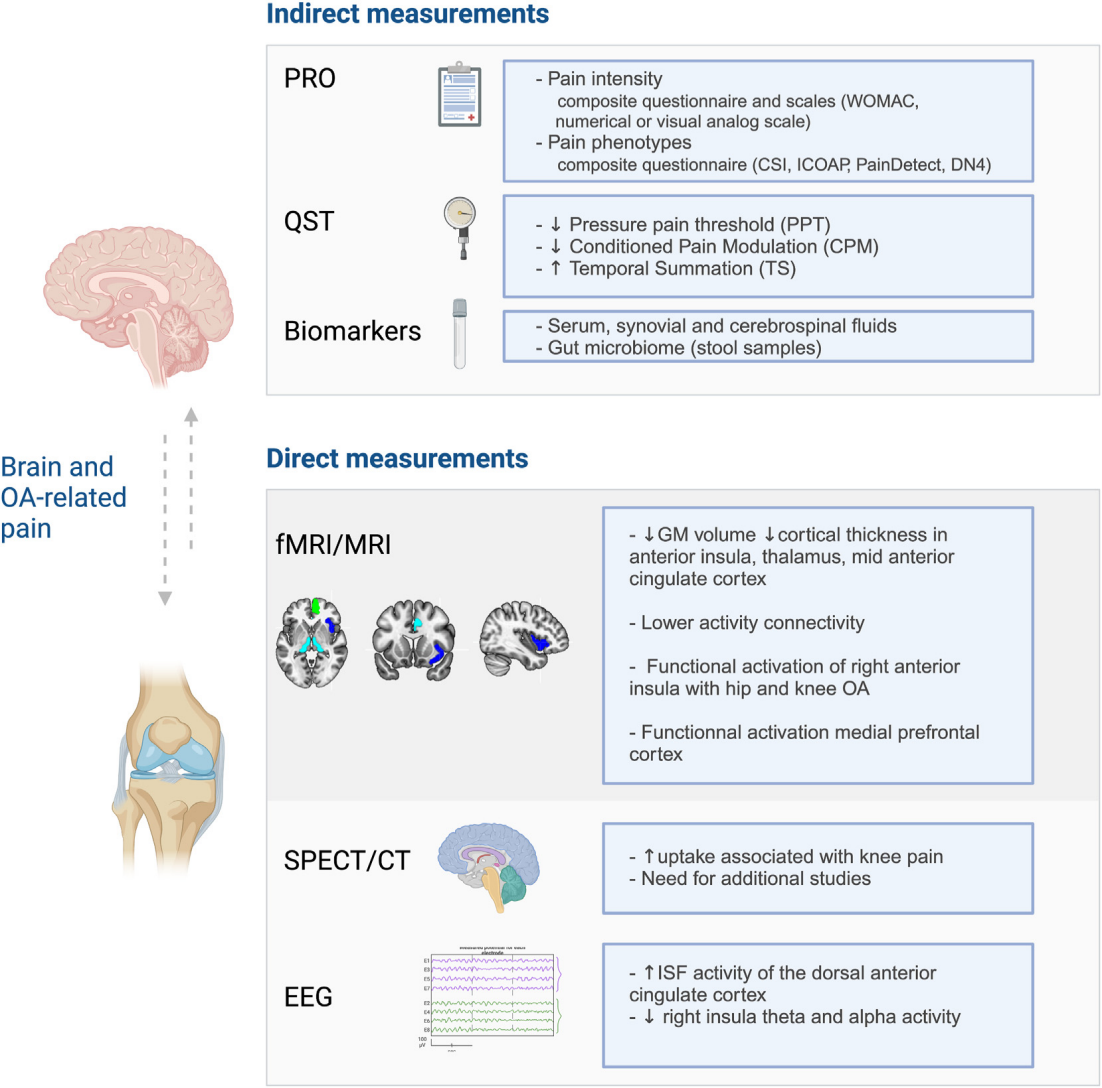
**Direct and indirect brain disturbances assessments associated with OA-related pain.** Indirect measurements include: i/Patient-Reported Outcome (PRO) with questionnaires such as the Western Ontario and McMaster Universities Arthritis Index (WOMAC) used in the evaluation of hip and knee OA; and composite questionnaires to identify pain phenotypes including the Central Sensitization Inventory - CSI; Intermittent and Constant Osteoarthritis Pain score – ICOAP; the neuropathic component assessed by the PainDetect and DN4 questionnaires. ii/Quantitative Sensory Testing (QST); and iii/Biomarkers. Direct measurements of brain activity state include: iv/Functional and anatomical MRI; v/Single-photon emission computed tomography or CT scan; and vi/electroencephalography (EEG).

In this sense, a few years ago was introduced the definition of nociplastic pain (i.e., pain that arises from altered nociception despite no clear evidence of actual tissue damage or lesion of the somatosensory system). One could argue that a part of the pain suggested to be neuropathic in the OA studies conducted at an earlier timepoint may be rather nociplastic. Nociplastic pain involves central sensitization due to neuroplastic changes in the nervous system in OA. Pain sensitization refers to pain induced by a non-painful stimulus (allodynia) or increased pain from as stimulus that normally provokes pain (hyperalgesia), with an enlarged painful area and longer duration of pain. In this phenomenon, central pain mechanisms are disturbed and characterized by an imbalance in the descending pain modulation pathway, including enhanced activity of pain facilitator, and loss of pain inhibitory circuits. Quantitative sensory testing (QST) measuring pain thresholds in various situations is used to identify the pain sensitization process, involved in the widespread pain phenomenon in OA and whose presence represents a critical component in post-operative pain following joint replacement [6]. However, QST provides only indirect clues to brain involvement in OA-related pain, while cerebrospinal fluid analysis, for example, is not easily feasible (Fig. 1).

Since the joint pain signal is processed by the nervous system from the peripheral nerves to the brain, feasible, non-invasive neuroimaging investigations are critical to provide a more direct assessment of brain disturbances associated with OA-related pain. Several neuroimaging and electrophysiological studies have shown that both neural and glial elements in sensory cortex, hypothalamus and midbrain, together with the dorsal horn of the spinal cord, show changes of central sensitization that modulate afferent nociceptive input and contribute to chronic pain in OA. The European multidisciplinary working group GO-PAIN (Going Inside Osteoarthritis-related Pain Phenotyping) has been launched to work on a global approach of the OA-related pain. A subcommittee from GO-PAIN, including 2 neuroscientists specialized in pain neuroimaging and 4 rheumatologists reviewed the available literature on brain neuroimaging in OA to provide a state-of-the-art view, discussion about current knowledge as well as field of improvement.

## 2 Brain imaging tests for chronic pain

Neuroimaging approaches such as MRI, positron emission tomography, single photon emission computed tomography (SPECT/CT), electroencephalography (EEG) and magnetoencephalography (MEG), are widely considered to have potential for diagnosis, stratification, and/or prediction of treatment outcomes in patients with chronic pain [7] including OA (Fig. 1). These non-invasive imaging technologies are used to produce three-dimensional detailed anatomical images of brain structures (e.g., T1-weighted MRI scans enable quantification of grey matter (GM) volume and cortical thickness (CT), and to determine changes in brain “*activation*” (i.e., fluctuation of BOLD – *blood-oxygen-level dependent*-signal in functional MRI) or “*connectivity*” between two brain regions (i.e., degree of synchronicity between different brain regions' activity) or at the level of groups of regions (e.g., network analysis) during various cognitive tasks. Such data offers advances in the understanding of brain organization and for assessing both structural and functional neurological status in relation with a disease or pain symptom.

Seminal works in brain imaging have illustrated that the process of subjective perception of pain (including sensory, affective and cognitive processes) involves the coordinated activation of multiple brain regions interacting dynamically [8], and is topologically organized in brain networks rather than a fixed arrangement of structures [9]. Brain regions of the so-called "pain matrix or network" are activated sequentially during the pain experience of pain [10]. The first brain activations associated with a pain stimulation mainly involved the posterior operculo-insular, mid cingulate cortex and the parietal sensory areas. This set of brain regions process raw nociceptive inputs from the ascending somatosensory pathway and the thalamus, but alone are not sufficient to sustain the conscious perception of pain. The involvement of higher-order set of regions belonging to cerebral networks outside this “nociceptive matrix” is crucial for nociception to become pain [8]. Second-order interactions link the raw sensory information with stimulus salience and motor control (I.e., primary and supplementary motor areas), pivotal areas in this task are the anterior insula and mid-anterior cingulate regions. The third order interactions link these sensorimotors and salience networks with brain structures that support high-level cognition and control including pain re-evaluation in prefrontal (e.g., orbito and dorso lateral prefrontal cortex) and posterior parietal regions (e.g., posterior cingulate cortex and precuneus. Limbic structure such as the amygdala-hippocampus regions are engaged early and encoding and is believed to be involved in the retrieval of emotionally charged memories associated with pain perception. The organization of these brain regions support the full-on conscious experience of pain [9,10].

Evidence supporting structural and functional changes in these brain networks in patients with chronic pain is mounting [11]. MRI measures of brain function that demonstrate aberrant pain mechanisms have shown the ability to classify individuals into unique brain activity maps associated with distinct chronic pain conditions [12]. Baliki and collaborators (2011), used structural brain MRI and illustrated that chronic back pain, complex regional pain syndrome and knee OA exhibited distinct grey matter density alterations compared to healthy control group. Decreased GM density in OA was localized to portions of the insula and mid anterior cingulate cortex in addition to the hippocampus, paracentral lobule and regions of the inferior temporal cortex. There is large overlapping between the three brain maps but also unique particularities that may be useful to identify subpopulations of patient and may contribute to the prediction of treatment such as ketamine therapy response in neuropathic pain [13]. This reflects the unique maladaptive physiology of different types of chronic pain and one of the main interests of using a complimentary neuroimaging approach in future studies in OA, which encompass a diversity of pain phenotypes.

## 3 What are the brain imaging data in the context of OA?

With proper methodology, brain imaging may offer objective biomarkers for OA-related pain and could potentially guide the development of novel personalized therapeutics or help optimization of use of current treatments. However, there is a general lack of understanding about how OA drives cortical changes in the pain circuit beyond joint tissue damage, and which brain mechanisms are involved in analgesic efficacy.

Some structural neuroimaging studies have identified GM distortions, distributed across cortical and subcortical structures in OA pain [14]. They mainly reported reduction in GM volume and lower CT in the anterior insula, thalamus, and mid anterior cingulate cortex [12,15,16]. OA pain has also been associated with widespread functional abnormalities in the activity and connectivity of brain networks involved in the processing and modulation of pain [17,18], without pointing out a specific set of regions.

In a recent well-conducted systematic literature review, Hall and colleagues (2023) synthetized results of neuroimaging studies on OA, including 26 studies [19]. This work has importance because it confirms the very high heterogeneity in reported results between OA and healthy controls, but also identifies some consistent key brain alterations associated with OA across experiments. Using an exploratory coordinate-based (Activation Likelihood Estimation- ALE) analysis including 18 experiments with various imaging methods (both structural (N ​= ​730 participants) and functional (N ​= ​457 participants) MRI studies), the authors found significantly lower activity, connectivity, and GM volume in the right anterior insula in patients with OA than in healthy controls. Consistent with these findings, the anterior insula was previously proposed as a key element driving changes in the brain of chronic OA pain patients [20]. Using graph theory with modular analysis on resting-state fMRI data, two studies have identified whole-brain network organization changes including alterations of connectivity in the anterior insula as well as other areas (para-hippocampal gyrus and parietal cortices) in OA pain [21,22].

The anterior insula is involved in many processes such as the ability to sense internal physiological and homeostatic condition of the body (*interoception*), but also the emotional experience and subjective feeling associated with nociception. The potentially lower right insular volume in OA compared to healthy controls might imply the dysfunction of this core region in the interoceptive awareness and the emotional context of sensory experience that contributes to OA pain. It is also well-known that the anterior insula is highly interconnected with sensory brain areas and therefore plays a central role in coordinating information on the physical-somatic nature of a noxious stimulus in the brain [9,23], which is why its alteration in OA could influence the perception of pain intensity and concomitant behavioral responses [24]. This interpretation is speculative and should be interpreted with caution since homogenous and larger neuroimaging studies are required in OA. However, its control over information passing between brain pain networks [25] is logically a locus of vulnerability, as has been reported for several brain disorders including other chronic pain conditions [26].

The anterior insula from the neuroimaging literature comparing OA and healthy controls emerged as the most consistently implicated region and associated with pain intensity. This finding indicate that dysregulated anterior insular activity represents a potential neurofunctional maladaptation in OA, but its involvement in the chronicity of OA pain remains unclear.

Beyond comparison between OA patients and controls, the site of OA may also influence neuroimaging features: the insula has been implicated in both knee and hip OA, but hip OA was associated with additional changes in the medial prefrontal cortex [19]. In addition, some studies found that the activity and/or connectivity of these two regions (anterior insula and orbital part of the prefrontal cortex) correlated with pain intensity (VAS) of OA patients [12,19,21,27,28], suggesting that activity of these two brain regions could serve as a potential surrogate biomarker. Brain imaging may also serve to decipher the mechanisms involved in placebo effect in OA: along this line, expectancy may reinforce the analgesic procedure in OA patients through activation of the medial prefrontal cortex and anterior cingulate cortex [29]. This contextual modulation of the pain experience may work through different pathways depending on the treatment modality, and probably, according to the pathophysiological states of the participants [30].

There have been several studies examining brain anatomical changes after joint replacement in OA. Abnormal structural properties of the brain's limbic circuitry (particularly the amygdala, hippocampus, together with the thalamus) and brainstem areas (such as the rostral ventromedial medulla) were associated with pain persistence after total arthroplasty [31,32]; whereas thalamic volume changes may reverse after successful arthroplasty and were associated with decreased pain and increased function [16]. These studies were motivated by the fact that the neural mechanisms for the persistence of pain after a technically successful arthroplasty in OA remain unclear. These findings demonstrate the presence of presurgical subcortical brain factors that relate to postsurgical persistence of OA pain. They challenge the view that mechanisms of OA pain predominantly underlie local joint mechanisms, implying to further include brain assessment to investigate OA pain, before or after joint replacement, and to improve pain characterization.

## 4 Effects of treatments on brain activity in OA

Several MRI works have investigated the effect of pharmacological and non-pharmacological treatments on brain activity in OA. The study of Lopez-Solà et al. (2022) [33] has shown that a single dose of naproxen reduces the brain activity in the pain neural circuit (“neurological pain signature”) compared to a placebo injection in OA. In line with previous studies [34], the effects of naproxen were located in the second somatosensory cortex, bilateral insula, basal ganglia, ACC, thalamus and amygdala [35]. Although these studies provide detailed insight about the brain regions that were modulated by naproxen over placebo, they lacked sufficient statistical power.

For non-pharmaceutical interventions, acupuncture is one of the most represented approaches. A recent review suggests that acupuncture-related therapy could regulate some brain regions in patients with knee OA [36]. Specifically, it showed that activity and connectivity changes were located in the prefrontal cortex, anterior cingulate cortex, insula, periaqueductal grey (PAG) and the limbic system including the hippocampus were involved in the regulation of OA during verum acupuncture compared to sham. These findings suggest that the beneficial effect of acupuncture could have a central impact on pain-related brain regions including the descending pain control pathway through the ACC, insula and PAG [37]. Pilot trials are currently investing in MRI the effect of non-invasive neuromodulation treatments (e.g. Ref. [38]).

Neuroimaging approaches may be used as complimentary measure of interest in clinical trials in OA to decipher mechanisms of pain alleviation or to predict the therapeutic response. Such measures are not intended to replace measures of patient-reported outcomes but rather serve as relevant neurobiological markers to track different outcomes. For example, physiological markers may be useful to confirm expected pharmacological effects on the physiological processes they are intended to target, and such results can then be used to make early stop/go decisions in clinical trials.

## 5 What are the limitations in the current evidence?

Although brain imaging techniques have revolutionized the study of the brain, there are several limitations. A practice issue of neuroimaging is its economic costs and constraints on their widespread implementation in clinical settings. Brain imaging is also limited by its ability to capture only a snapshot of the brain at a particular moment in time, and it is difficult to capture the dynamic changes that occur in the brain over time. To encompass this limitation, multimodal neuroimaging at different brain scale may be recorded using resting-state as well as task-based experimental designs [39]. It may help to develop composite biomarkers (see [40]). Brain imaging can also produce false positives caused by artifacts or noise in the images or by individual variations in brain structure or function. Therefore, it is mandatory to follow recommendations [7], correct and test for inter-individual differences (e.g., age, sex, psychological states, etc.).

Despite an increasing number of brain imaging studies in OA-related pain, most of them compared patients with OA and healthy controls but did not investigate specific changes between symptomatic and non-symptomatic OA patients, which could give more precise insights into brain alterations associated with chronic OA-related pain. In addition, interpretation of many studies is limited by the heterogeneity of methods, small sample sizes, and the absence of validation studies. Furthermore, although some differences have been found between OA and rheumatoid arthritis or fibromyalgia, the specificity of such brain disturbances between painful diseases needs to be addressed [41,42].

OA pain patients have also altered pain thresholds in terms of pain sensitization assessed by QST [6,43]. Neuroimaging arguments indicate that this central sensitization phenomenon could be associated with a disruption of central pain inhibitory pathways, including greater activation in the PAG matter in response to mechanical stimuli [44,45]. The sensitization phenomenon was also found to be associated with brain changes extending beyond strict pain-processing regions with enhancement of activity in general sensory, and non-nociceptive brain areas [41,46]. However, the link between brain changes and clinical readouts (Fig. 1) such as QST measures or PROs assessing neuropathic or nociplastic pain phenotypes in OA has never really been addressed [19].

One challenge in imaging chronic OA pain is also to consider patient variability (e.g., disease severity in terms of structural damages and number of OA sites, disease duration, ongoing medication, depression and anxiety) and factors influencing findings’ specificity such as sex differences [47]. Unfortunately, their influence has not been studied in the current literature. For instance, it seems that brain alterations may change according to sex in murine model of OA [48]. The effects of sex on brain patterns have been studied in chronic pain and are now considered a crucial factor to test, but to our knowledge, no specific studies on human OA have been conducted to date (see for more details [49]).

Recent studies have pointed out factors that may influence brain activity such neuroinflammation and sleep quality. Pain-related sleep interference (insomnia) in patients with OA contributes to enhanced pain sensitivity, as measured by quantitative sensory testing [[50], [51], [52]]. There is a close interaction between sensory hypersensitivity and sleep disturbances: a single night of total sleep deprivation has been shown to induce generalized hyperalgesia and increase state anxiety in healthy people [53], and sleep curtailment impairs endogenous nociceptive-inhibitory function and increases spontaneous pain [54]. This may ensue a vicious cycle: poor sleep lowers pain thresholds, which then contributes to hyperalgesia and subsequent increased incidence and/or severity of insomnia. The vast majority of trials addressing symptomatic knee OA do not capture sleep measures, and future research should include formal sleep-centric assessments measured at multiple time points to analyze sleep dysfunction and its relationship on treatment effects [55].

In patients with knee OA, elevated cerebrospinal fluid levels of 48 proteins, including monocyte chemoattractant protein 1 and interleukin-8, have been observed, suggesting the presence of neuroinflammation in these patients [56,57]. Interestingly, seven of the 48 elevated cerebrospinal fluid proteins have established neuroprotective effects, and are associated with lower pain intensity and milder knee-related symptoms in patients with knee OA.

All these factors contribute to analysis bias. Moreover, to study the chronicity of brain disturbances, future studies would also need to consider longitudinal follow-up, preferably before and after analgesic treatment.

Beyond these scientific unmet needs, identification of brain-based markers of OA requires more standardized technological approaches, higher sample size with large-scale data acquisition across diverse cohorts and independent group of individuals for validation, and strict application of standards of evidence [7]. In this way, recent brain network analysis has shown promising results to reveal the broader context of network abnormalities due to chronic OA pain in both sexes; and to identify the set of brain regions that reliably predicted pain intensity in knee OA; this result generalized to hip OA [21].

In conclusion, brain functional imaging provides opportunities to accurately address an OA-related pain endophenotype [1]. To encompass limitations and fill the gaps from the previous studies, we propose a blueprint for the future (Table 1). Functional neuroimaging investigations will surely improve our understanding of OA-related pain and could delineate several profiles of painful patients. Nonetheless, these kinds of investigations will hold relevance only within a comprehensive strategy that integrates a multimodal assessment, combining PROs, joint imaging, QST, patient characteristics and qualitative experience of pain. Such a challenge is especially relevant, considering the emerging field of non-invasive brain stimulation (rTMS and tDCS) as a potential therapy for OA-related pain sensitization [58], that could open toward implementation of brain imaging in clinical practice to personalize therapeutic management.

**Table 1:** Research agenda for brain imaging investigations in OA-related pain.

(1) Areas for improvement in study design • Discovery and validation cohorts • Longitudinal assessment • Adequate sample size of patient with homogeneous OA conditions • Adequate comparator group (eg non-painful OA patients instead of healthy controls or OA patients with distinct pain phenotypes) • Consideration of bias of analysis related to the patients or to the disease (e.g. sex differences, characteristics of the disease, demographic, medications, comorbidities, disease severity, diffuse OA)
(2) **N**euroimaging improvements • Improvement of brain neuroimaging techniques (e.g., glial imaging in TEP; sophisticated approach with multimodal imaging – temporal, spatial and spectral - to characterize brain networks topological organization and dynamics at multiple scales in the aim to isolate composite biomarkers) • Standardization of brain neuroimaging technics across studies • Use advanced brain imaging approaches such as connectomics, functional brain ultrasound, deep learning and the ones targeting glial physiology in patients with OA • To create a neuroimaging repository of brain imaging data in OA, where pain researchers can share their data
(3) **Sc**ientific questions and perspectives • To identify brain changes (morphological, functional) associated with specific pain phenotype in people with OA pain (eg nociceptive vs nociplastic OA pain) or with central neuroinflammation features • To identify brain changes related to the number of OA painful joints • To specifically assess the effect of sex differences on brain neuroimaging • Evaluating and/or predicting the efficacy of treatments for pain • Exploring brain-targeting therapies such as non-invasive neuromodulation therapy (efficacy and mechanism of action) • Implementation of brain neuroimaging in clinical practice to personalize OA pain management

## Author contributions

CF and JS drafted the manuscript; MB, FB, PC and RP edited the manuscript; all authors approved the final manuscript.

## Role of the funding source

NEURON-ERA-NET (Network of European Funding for Neuroscience Research) and Agence Nationale de la Recherche (ANR) that fund the GO-PAIN networks (for organization and logistical requirments for the network).

## Declaration of competing interest

CF and RP report no competing interest.

MB's research is funded by a grant from the French Society of Rheumatology, the Osteoarthritis Foundation grant, Pfizer Advance 2020 grant, and a doctoral fellowship from Sorbonne University.

JS reports personal fees from MSD, Pfizer, Abbvie, Fresenius Kabi, BMS, Roche, Chugai, Sandoz, Lilly, Novartis, Galapagos, AstraZeneca, UCB, Grunenthal and Janssen and research grants from Pfizer, Schwa Medico, and BMS.

FB received an institutional grant from TRB Chemedica and Pfizer and consulting fees from AstraZeneca, Boehringer Ingelheim, Bone Therapeutics, Cellprothera, Galapagos, Gilead, Grunenthal, GSK, Lilly, MerckSerono, MSD, Nordic Bioscience, Novartis, Pfizer, Roche, Sandoz, Sanofi, Servier, UCB, Peptinov, 4P Pharma, and 4Moving Biotech.

PC has done speakers bureaus or consultancies for AbbVie, AstraZeneca, Eli Lilly, Eupraxia, Galapagos, Genascence, GSK, Grunenthal, Janssen, Levicept, Medipost, Merck, Moebius, Novartis, Sandoz, Stryker, TrialSpark and UCB.

## Appendix

Going Inside Osteoarthritis-Related Pain (GO-PAIN) network members: Françoise Alliot-Launois, Nadine Attal, Francis Berenbaum, Marie Binvignat, Philip Conaghan, Alice Courties, Niels Eijkelkamp, Camille Fauchon, Nathan Foulquier, Rinie Geenen, Ida K Haugen, Yves Henrotin, Kalle Kisand, Margreet Kloppenburg, Eva Kosek, Liisa Kuhi, Sylvain Mathieu, Céline Mathy, Ali Mobasheri, Stanislas Moumbe Talla, Patrick Omoumi, Serge Perrot, Roland Peyron, Simo Saarakkala, Alain Saraux, Hans-Georg Schaible, Jérémie Sellam.

## References

[1] Saxer F. (2024). Pain-phenotyping in osteoarthritis: current concepts, evidence, and considerations towards a comprehensive framework for assessment and treatment. Osteoarthr Cartil Open.

[2] Hunter D.J., Bierma-Zeinstra S. (2019). Osteoarthritis. Lancet.

[3] Conaghan P.G. (2019). Therapeutic options for targeting inflammatory osteoarthritis pain. Nat. Rev. Rheumatol..

[4] Berenbaum F. (2013). Osteoarthritis as an inflammatory disease (osteoarthritis is not osteoarthrosis!). Osteoarthritis Cartilage.

[5] Dimitroulas T. (2014). Neuropathic pain in osteoarthritis: a review of pathophysiological mechanisms and implications for treatment. Semin. Arthritis Rheum..

[6] Arant K.R., Katz J.N., Neogi T. (2022). Quantitative sensory testing: identifying pain characteristics in patients with osteoarthritis. Osteoarthritis Cartilage.

[7] Davis K.D. (2017). Brain imaging tests for chronic pain: medical, legal and ethical issues and recommendations. Nat. Rev. Neurol..

[8] Garcia-Larrea L., Bastuji H. (2018). Pain and consciousness. Prog. Neuro Psychopharmacol. Biol. Psychiatr..

[9] Fauchon C. (2020). The modular organization of pain brain networks: an fMRI graph analysis informed by intracranial EEG. Cerebral Cortex Communications.

[10] Bastuji H. (2016). Pain networks from the inside: spatiotemporal analysis of brain responses leading from nociception to conscious perception: pain Networks from the inside. Hum. Brain Mapp..

[11] Xu A. (2021). Brain responses to noxious stimuli in patients with chronic pain: a systematic review and meta-analysis. JAMA Netw. Open.

[12] Baliki M.N. (2011). Brain morphological signatures for chronic pain. PLoS One.

[13] Bosma R.L. (2018). Brain dynamics and temporal summation of pain predicts neuropathic pain relief from ketamine infusion. Anesthesiology.

[14] Barroso J. (2020). Brain gray matter abnormalities in osteoarthritis pain: a cross-sectional evaluation. Pain.

[15] Alshuft H.M. (2016). Cerebral cortical thickness in chronic pain due to knee osteoarthritis: the effect of pain duration and pain sensitization. PLoS One.

[16] Gwilym S.E. (2010). Thalamic atrophy associated with painful osteoarthritis of the hip is reversible after arthroplasty: a longitudinal voxel-based morphometric study. Arthritis Rheum..

[17] Iwabuchi S.J. (2023). Medio-dorsal thalamic dysconnectivity in chronic knee pain: a possible mechanism for negative affect and pain comorbidity. Eur. J. Neurosci..

[18] Kulkarni B. (2007). Arthritic pain is processed in brain areas concerned with emotions and fear. Arthritis Rheum..

[19] Hall M. (2023). Neurobiology of osteoarthritis: a systematic review and activation likelihood estimation meta-analysis. Sci. Rep..

[20] Cottam W.J. (2018). Altered connectivity of the right anterior insula drives the pain connectome changes in chronic knee osteoarthritis. Pain.

[21] Barroso J. (2021). Reorganization of functional brain network architecture in chronic osteoarthritis pain. Hum. Brain Mapp..

[22] Mansour A. (2016). Global disruption of degree rank order: a hallmark of chronic pain. Sci. Rep..

[23] Bastuji H. (2018). Convergence of sensory and limbic noxious input into the anterior insula and the emergence of pain from nociception. Sci. Rep..

[24] Blair R.J. (2016). The neurobiology of impulsive aggression. J. Child Adolesc. Psychopharmacol..

[25] Joyce K.E. (2010). A new measure of centrality for brain networks. PLoS One.

[26] van den Heuvel M.P., Sporns O. (2013). Network hubs in the human brain. Trends Cognit. Sci..

[27] Cottam W.J. (2016). Associations of limbic-affective brain activity and severity of ongoing chronic arthritis pain are explained by trait anxiety. Neuroimage Clin.

[28] Ushio K. (2020). Altered resting-state connectivity with pain-related expectation regions in female patients with severe knee osteoarthritis. J. Pain Res..

[29] Kong J. (2018). Enhancing treatment of osteoarthritis knee pain by boosting expectancy: a functional neuroimaging study. Neuroimage Clin.

[30] Gollub R.L. (2018). A functional neuroimaging study of expectancy effects on pain response in patients with knee osteoarthritis. J. Pain.

[31] Barroso J. (2023). Subcortical brain anatomy as a potential biomarker of persistent pain after total knee replacement in osteoarthritis. Pain.

[32] Soni A. (2019). Central sensitization in knee osteoarthritis: relating presurgical brainstem neuroimaging and PainDETECT-based patient stratification to arthroplasty outcome. Arthritis Rheumatol..

[33] López-Solà M. (2022). The neurologic pain signature responds to nonsteroidal anti-inflammatory treatment vs placebo in knee osteoarthritis. Pain Rep.

[34] Giménez M. (2014). Naproxen effects on brain response to painful pressure stimulation in patients with knee osteoarthritis: a double-blind, randomized, placebo-controlled, single-dose study. J. Rheumatol..

[35] Sanders D. (2015). Pharmacologic modulation of hand pain in osteoarthritis: a double-blind placebo-controlled functional magnetic resonance imaging study using naproxen. Arthritis Rheumatol..

[36] Qu Y. (2024). Acupuncture-related therapy for knee osteoarthritis: a narrative review of neuroimaging studies. J. Pain Res..

[37] Zhou J. (2023). Modulation effects of different treatments on periaqueductal gray resting state functional connectivity in knee osteoarthritis knee pain patients. CNS Neurosci. Ther..

[38] Drabek M. (2023). Brain connectivity-guided, Optimised theta burst transcranial magnetic stimulation to improve Central Pain Modulation in knee Osteoarthritis Pain (BoostCPM): protocol of a pilot randomised clinical trial in a secondary care setting in the UK. BMJ Open.

[39] Fauchon C., Di Ieva A. (2024). The Fractal Geometry of the Brain.

[40] Zhang L.-B. (2024). Advances and challenges in neuroimaging-based pain biomarkers. Cell Reports Medicine.

[41] Pujol J. (2022). Distinctive alterations in the functional anatomy of the cerebral cortex in pain-sensitized osteoarthritis and fibromyalgia patients. Arthritis Res. Ther..

[42] Sundermann B. (2019). Subtle changes of gray matter volume in fibromyalgia reflect chronic musculoskeletal pain rather than disease-specific effects. Eur. J. Neurosci..

[43] Suokas A.K. (2012). Quantitative sensory testing in painful osteoarthritis: a systematic review and meta-analysis. Osteoarthritis Cartilage.

[44] Gwilym S.E. (2009). Psychophysical and functional imaging evidence supporting the presence of central sensitization in a cohort of osteoarthritis patients. Arthritis Rheum..

[45] Iwabuchi S.J. (2020). Brain perfusion patterns are altered in chronic knee pain: a spatial covariance analysis of arterial spin labelling MRI. Pain.

[46] Pujol J. (2017). Brain imaging of pain sensitization in patients with knee osteoarthritis. Pain.

[47] Fauchon C. (2021). Sex differences in brain modular organization in chronic pain. Pain.

[48] Da Silva J. (2021). Pain modulatory network is influenced by sex and age in a healthy state and during osteoarthritis progression in rats. J. Pain.

[49] Gupta A. (2017). Sex-based differences in brain alterations across chronic pain conditions. J. Neurosci. Res..

[50] Bjurström M.F. (2021). Preoperative sleep quality and adverse pain outcomes after total hip arthroplasty. Eur. J. Pain.

[51] Parmelee P.A., Tighe C.A., Dautovich N.D. (2015). Sleep disturbance in osteoarthritis: linkages with pain, disability, and depressive symptoms. Arthritis Care Res..

[52] Campbell C.M. (2015). Sleep, pain catastrophizing, and central sensitization in knee osteoarthritis patients with and without insomnia. Arthritis Care Res..

[53] Schuh-Hofer S. (2013). One night of total sleep deprivation promotes a state of generalized hyperalgesia: a surrogate pain model to study the relationship of insomnia and pain. Pain.

[54] Smith M.T. (2007). The effects of sleep deprivation on pain inhibition and spontaneous pain in women. Sleep.

[55] Feda J. (2023). Measures of sleep are not routinely captured in trials assessing treatment outcomes in knee osteoarthritis - a scoping systematic review and call to action. Osteoarthr Cartil Open.

[56] Kosek E. (2018). Differences in neuroimmune signalling between male and female patients suffering from knee osteoarthritis. J. Neuroimmunol..

[57] Palada V. (2020). Elevated inflammatory proteins in cerebrospinal fluid from patients with painful knee osteoarthritis are associated with reduced symptom severity. J. Neuroimmunol..

[58] Dissanayaka T. (2023). The effects of anodal tDCS on pain reduction in people with knee osteoarthritis: a systematic review and meta-analysis. Neurophysiol. Clin..

